# Weight dependence in BCM leads to adjustable synaptic competition

**DOI:** 10.1007/s10827-022-00824-w

**Published:** 2022-06-29

**Authors:** Albert Albesa-González, Maxime Froc, Oliver Williamson, Mark C. W. van Rossum

**Affiliations:** School of Psychology and School of Mathematical Sciences, University of Nottingham, Nottingham, NH7 2RD UK

**Keywords:** Synaptic plasticity, BCM, learning rule, STDP

## Abstract

Models of synaptic plasticity have been used to better understand neural development as well as learning and memory. One prominent classic model is the Bienenstock-Cooper-Munro (BCM) model that has been particularly successful in explaining plasticity of the visual cortex. Here, in an effort to include more biophysical detail in the BCM model, we incorporate 1) feedforward inhibition, and 2) the experimental observation that large synapses are relatively harder to potentiate than weak ones, while synaptic depression is proportional to the synaptic strength. These modifications change the outcome of unsupervised plasticity under the BCM model. The amount of feed-forward inhibition adds a parameter to BCM that turns out to determine the strength of competition. In the limit of strong inhibition the learning outcome is identical to standard BCM and the neuron becomes selective to one stimulus only (winner-take-all). For smaller values of inhibition, competition is weaker and the receptive fields are less selective. However, both BCM variants can yield realistic receptive fields.

## Introduction

One of the hallmarks of the nervous system is it’s adaptive character. Over long time-scales the responses of neurons adapt to the input that it receives. On the neural level the adaptive character is perhaps nowhere more clearly observed than in the primary visual cortex. Manipulation of the visual environment and closure of the eyes have been shown to strongly affect the development of the visual system and the receptive fields that emerge (Wiesel & Hubel, 1963). For instance, under normal conditions neurons in binocular cortex receive inputs from both retinas and become responsive to inputs from both eyes. However, when one eye is closed during development it loses its drive onto the neurons. Synaptic changes are believed to be responsible for this type of adaptation.

To describe such neurophysiological experiments on the development of the visual cortex, the Bienenstock, Cooper and Munro (BCM) model was introduced some 40 years ago as a computational theory of synaptic plasticity (Bienenstock et al., 1982; Clothiaux et al., 1991). BCM theory is a unsupervised learning rule that contains two important ingredients: First, as in other Hebbian models of synaptic plasticity, high post-synaptic activity increases (potentiates) synaptic strengths of inputs that are co-active, while low post-synaptic activity leads to a weakening (depression) of synaptic strength of active inputs. However, this could lead to exponential run-away plasticity as strengthened synapses are more likely to lead to strong responses and will be subsequently potentiated further. Therefore a second ingredient, unique to BCM, is that the threshold that determines the switch-point between depression and potentiation is adjusted depending on the running average activity of the post-synaptic neuron. When the activity of the neuron becomes too high, the threshold for potentiation is increased so that synapses will become more likely to be depressed. As a result, with the right parameters stable receptive fields develop in the BCM model without the need for additional bounds on the synaptic weights or other homeostatic mechanisms.

The BCM model has stood the test of time and remains one of the leading models for unsupervised cortical plasticity (reviewed in Cooper & Bear, 2012). While BCM is not a microscopic model of plasticity, over the years the connection to more microscopic details has been strengthened, for instance by incorporation of the role of calcium influx (Shouval et al., 2002b) and linking BCM to Spike Timing Dependent Plasticity (Izhikevich & Desai , 2003; Graupner & Brunel, 2012; Gjorgjieva et al., 2011).

The BCM model typically forms highly selective receptive fields so that the neuron after learning is active only in response to one or a few input patterns. However, with some tweaks the BCM model can also be modified to learn receptive fields such as found in primary visual cortex.

In an effort to incorporate more biological detail, we include here two effects. First, under standard BCM the synapses change sign, which is at odds with biology. Therefore we split the synaptic inputs into excitatory and inhibitory ones. Under general conditions this by itself does not change the standard BCM model (Scofield & Cooper, 1985), but it becomes important when we include the second modification, namely the dependence of plasticity on the synaptic strength. It has been observed that the relative amount of long term potentiation is less for strong synapses than for weak synapses (Debanne et al., 1999; Montgomery et al., 2001; Loebel et al., 2013). Meanwhile, the percentage decrease in strength appears to be independent of strength itself when synaptic depression protocols are used (Debanne et al., 1996; Bi & Poo, 1998). The phenomenon is known as soft-bound or weight dependent plasticity. Indirect evidence for soft-bound plasticity stems from the central distribution of synaptic weights (e.g. Zhang et al., 2015), which follows naturally from soft-bound plasticity.

Here we study how inclusion of weight dependence in BCM plasticity combined with feed-forward inhibition changes the outcome of learning. We find that the strength of inhibition determines the selectivity that develops, but in the limit of strong inhibition one recovers results from standard BCM.

## Weight dependent BCM

### Definition of the standard BCM model

**Fig. 1:**
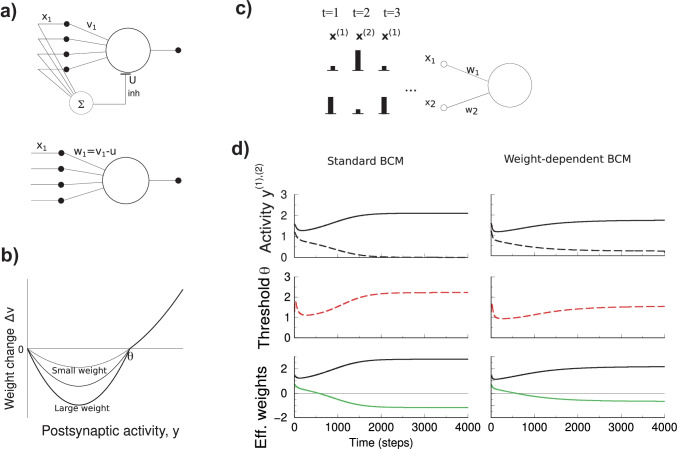
Weight dependent BCM model and its dynamics for a neuron with two inputs. **a**. Top: Neuron receiving input through excitatory weights and parallel fixed feed-forward inhibition. Bottom: Reduction to a neuron with effective weights $w_{i}$, that combine the excitatory weights $v_{i}$ and the fixed inhibition *u*, so that $w_{i}=v_{i}-u$. **b**. In weight dependent BCM the depression part of modification curve depends on the weight itself. When the weight is large, depression is strong (thick curve); while for weak weights depression is limited (thin curve). The potentiation part of the curve is unmodified. **c**. Setup of the 2D system. The input vector alternates between $\varvec{x}^{(1)}=(\cos \phi ,\sin \phi )$ and $\varvec{x}^{(2)}=(\sin \phi ,\cos \phi )$. The superscript labels the stimulus. **d**. Left: Example result of a simulation of standard BCM using the stimulation protocol of panel c. After an initial transient the system finds a stable fixed point. From top to bottom: the post-synaptic activity *y* in response to patterns 1 (solid) and 2 (dashed), the plasticity threshold $\theta$, and the evolution of the synaptic weights $w_{1}$ and $w_{2}$ (black and green). In standard BCM the final weight configuration is strongly selective, as the post-synaptic activity becomes zero for one input pattern and high for the other input pattern. Right: In the weight dependent BCM model the dynamics is similar but the fixed points leads to less selective post-synaptic activation (compare dashed curve in top panels). (Stimulus angle $\phi =0.4;$ inhibition $u=1.3$)

We consider a neuron with *N* modifiable synapses whose strength is coded in the weight vector $\varvec{w}=(w_{1},\ldots ,w_{N})$, Fig. 1a. The inputs are denoted with the vector $\varvec{x}$. As the entries in $\varvec{x}$ represent firing rates we assume them positive, $x_{i}\ge 0$. The post-synaptic neuron driven by these inputs has an activity $y=g(\varvec{w\cdot x})$. Here *g*() is the neuron’s input-output transfer function.

The standard BCM synaptic modification rule is defined as follows. First, the weights are updated according to

1 $$\begin{aligned} \tau _{w}\frac{dw_{i}}{dt} & =x_{i}F(y) \\ & = x_i y(y-\theta) \end{aligned}$$

where $\tau _{w}$ determines the update rate of the synapses, and $F(y)=y(y-\theta )$ is a function of the post-synaptic activity.

By itself this plasticity rule can lead to run-away divergence of synaptic weights. A synapse will be increased when $y>\theta$, but strong weights lead to more post-synaptic activity and hence more potentiation. Likewise, very low activity will lead to depression of already weak weights. Therefore the threshold $\theta$ dynamically tracks the average post-synaptic activity squared with a time-constant $\tau _{\theta }$, so that the condition for potentiation becomes harder as post-synaptic activity increases,

2 $$\begin{aligned} \tau _{\theta }\frac{d\theta }{dt}=-\theta +y^{2} \end{aligned}$$

This is the second ingredient of BCM. Experimental evidence for such a dynamical shift of the threshold shift has been observed (Kirkwood et al., 1996; Lim et al., 2015).

Together, the weights and threshold updates of the BCM model form a dynamical system driven by the input patterns, and with $\tau _{w}$ and $\tau _{\theta }$ as parameters. The dynamical repertoire of the standard BCM model has recently been studied in detail (Udeigwe et al., 2017). To catch run-away plasticity the threshold update needs to be supra-linear in the activity ($y^{2}$-term in Eq. (2)). The threshold update also needs to be fast enough, otherwise the system becomes unstable and strong oscillations in the synaptic weights or chaos result. On the other hand, the update needs to be slow enough to capture the average activity.

Typically the stimuli $\varvec{x}$ are drawn from a set of *K* stimuli. A given stimulus is indexed with a superscript $\varvec{x}^{(k)}$. With $y^{(k)}$ we denote the response of the neuron to that stimulus. Assuming that every unit time-step a new pattern is presented, we require that the threshold represents the average activation but also that updates are small, i.e. we require $1/K\ll \tau _{\theta }\ll \tau _{w}$. When $\tau _{w},\tau _{\theta }$ are slow enough we can replace the threshold by its mean field average over the stimuli $\theta =\frac{1}{K}\sum _{k}\left[ y^{(k)}\right] ^{2}$. In other words, the dynamical system becomes *N*-dimensional (Udeigwe et al., 2017).

### Inhibition in BCM models

In the standard BCM model the synaptic weights can be excitatory or inhibitory and can change sign. This is at odds with biology. While during early development GABA receptors can change from excitatory to inhibitory (Owens et al., 1996), this capacity is later lost and commonly not believed to be part of ongoing plasticity. Yet, inhibition is indispensable in order to obtain selective receptive fields. To allow for effectively negative weights, we adopt the common solution that plastic excitatory connections exist in parallel to feed-forward inhibitory connections, Fig. 1a. The inhibitory neuron pools the inputs and inhibits the excitatory neuron proportionally. Denoting the excitatory weights as $v_{i}$ and the uniform inhibitory strength *u*, the net input to the neuron is $h=\sum _{i}v_{i}x_{i}-u\sum _{i}x_{i}=\sum _{i}(v_{i}-u)x_{i}$. We can identify the* effective* *weights* as $w_{i}=v_{i}-u$, which are thus constrained as $w_{i}\ge -u$.

The plasticity has to be distributed over the excitatory and inhibitory connections. For instance one could make inhibition plastic and keep excitation fixed. Here, however, we keep the inhibitory connections fixed and update the excitatory weights as in Eq. (1), hence $\Delta v_{i}=\Delta w_{i}$. The plasticity of the excitatory connections thus happens on a background of fixed inhibition.

As long as the excitatory weights do not reach their minimum bound of 0, the model behaves mathematically exactly like the standard BCM model. So, in the standard BCM model splitting the effective weights into plastic excitatory weights and fixed inhibitory weights has no consequence on the outcome of plasticity, as was already analyzed in Scofield and Cooper (1985).

### The weight dependent BCM model

However, the split into excitatory and inhibitory connections does matter when we include weight dependence of plasticity. In experiments it has been observed that plasticity depends on the current weight of the synapse. As typical plasticity experiments use extracellular stimulation and recording, one does not know how many synapses are being stimulated. Therefore it is common to report the relative changes in synaptic strength, which should be distinguished from the absolute amounts of plasticity used in the model. It has been observed that this relative amount of long term potentiation is smaller for already strong synapses than for weak synapses (Debanne et al., 1999; Montgomery et al., 2001; Loebel et al., 2013; Zhang et al., 2015). Meanwhile, for depression protocols the percentage decrease in strength appears to be independent of strength itself (Debanne et al., 1996).

To implement weight-dependence of the plasticity in BCM, we modify the learning rule as follows. When potentiation occurs, the original BCM rule Eq. (1) still applies as before. However, whenever the synapse is depressed, the excitatory weight is depressed with an amount proportional to the excitatory weights, i.e. $\frac{dv_{i}}{dt}\propto v_{i}$. When the rule is expressed in the effective weights $w_{i}$ one has

3 $$\begin{aligned} \tau _{w}\frac{dw_{i}}{dt} ={\left\{ \begin{array}{ll} (w_{i}+u)\,x_{i}y(y-\theta ) &{} \text {if }y(y-\theta )<0\text { (depression),}\\ x_{i}y(y-\theta ) &{} \text {otherwise (potentiation)}. \end{array}\right. } \end{aligned}$$

Note that indeed in case of depression the relative amount of change in the excitatory synapse, $\Delta v_{i}/v_{i}={\Delta }{w_{i}/(w_{i}+u)}$, is independent of the weight, while for potentiation $\Delta v_{i}/v_{i}\propto 1/v_{i}$, as has been observed experimentally. The threshold update, Eq. (2) is unaltered in the weight dependent BCM model. The resulting modification curve of weight dependent BCM is sketched in Fig. 1b. Weight dependence has also been observed in spike timing dependent plasticity (STDP) protocols (Bi & Poo, 1998). In the appendix we explain how weight-dependence can be included in STPD-based BCM models and how this can lead to the above model.

Mathematically, should an excitatory synapse become inhibitory ($w_{i}<-u$), the model’s definition ensures that it will only experience potentiation and quickly become excitatory again. Provided $x_{i}\ge 0$, the weight dependent BCM model automatically obviates the need for hard bounds, which aids analysis.

## Outcome of BCM with two inputs

In order to gain intuition in the weight dependent BCM model we start with a neuron with just two inputs that is stimulated with two alternating patterns. The patterns are denoted as vectors $\varvec{x}^{(k)}$, where the superscript $k=1..K$ indicates the presented pattern. Following Udeigwe et al. (2017) we use the parametrization $\varvec{x}^{(1)}=(\cos \phi ,\sin \phi )$ and $\varvec{x}^{(2)}=(\sin \phi ,\cos \phi )$. These are vectors with unit length mirrored in (1, 1) and an angle $\pi /2-2\phi$ between them.

We set $\phi =0.4$, initialize with small weights and simulate until the weights equilibrate, Fig. 1c. In preliminary simulations of weight dependent BCM we found that the parameters $\tau _{\theta }$ and $\tau _{w}$ required for stability were similar to those needed for standard BCM. As the threshold needs to average all *K* inputs and $K=N$, we typically used $\tau _{\theta }=10N$, $\tau _{w}=10\tau _{\theta }$. Provided the system is stable, the fixed points won’t depend on these parameters.

Code was implemented in Octave with a C routine for efficient simulation of BCM, and is available at https://github.com/vanrossumlab/weight-dependent-BCM. The neuron was linear $y=\varvec{w \cdot x}$. We also tried a rectifying non-linearity, however, as the activity typically doesn’t become negative this lead to very similar results.

### Standard BCM for a neuron with two inputs

When the classical BCM model is repeatedly presented with two alternating stimuli, the synaptic weights develop to a stable value. More precisely, there are two stable fixed points (Castellani et al., 1999; Cooper et al., 2004; Udeigwe et al., 2017). In general the fixed points of the learning dynamics are the weights at which the average update is zero, i.e. $\langle \Delta \varvec{w}\rangle =\varvec{0}$, where the brackets denote the average over the stimuli. Using that the stimulus vectors are linearly independent in Eq. (1) leads to the stronger condition that the weight change in response to each stimulus is zero, i.e. $\Delta \varvec{w}^{(1)}=\Delta \varvec{w}^{(2)}=0$. Hence either $y^{(k)}=0$ or $y^{(k)}=\theta$. As there are two input patterns, there are four cases to consider. It turns out that the fixed point is stable when for one input pattern the output $y^{(1)}=\theta$, and for the other input pattern the neuron remains silent $y^{(2)}=0$ (or vice versa). The cases $y^{(1)}=y^{(2)}=0$, or $y^{(1)}=y^{(2)}=\theta$ are also fixed points, but are unstable. Writing the stimuli as a square matrix *X*, so that $y^{k}=\sum _{i}X_{k,i}w_{i}$, the stable fixed points are

4 $$\begin{aligned} w_{i}=2(X^{-1})_{i,m} \end{aligned}$$

where $m=1,2$ indexes either fixed point.

Simulation confirms these classic results, Fig. 1d. After learning has stabilized, the neuron is active in response to one particular input and falls silent in response to the other, Fig. 1d (left, top). The initial conditions determine which stimulus wins. Thus under standard BCM the neuron develops to become highly selective (winner-take-all competition).

### Weight dependent BCM for a neuron with two inputs

**Fig. 2:**
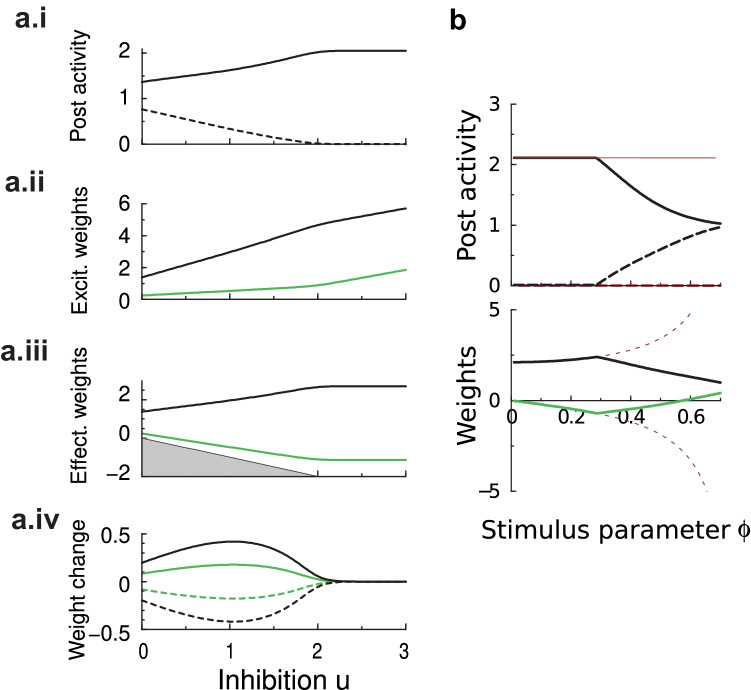
The fixed points of weight dependent BCM as a function of the amount of feed-forward inhibition for a neuron with two inputs. **a**. Outcome of weight dependent BCM as a function of the strength of the feed-forward inhibition after plasticity has equilibrated. Showing from top to bottom: i) the post-synaptic activity *y* to input pattern 1 (solid) and 2 (dashed). For low inhibition, the neuron becomes weakly selective. For strong inhibition (right region, $u\gtrsim 2$), the outcome is identical to the standard BCM result and has winner-take-all selectivity. ii) The excitatory weights $v_{1,2}$ (weight 1: black; weight 2: green). iii) The effective weights $w_{1,2}$. The grey area indicates a forbidden region where the excitatory weight would be negative. iv) The amount of change in the weights in response to either input pattern (the smaller weight is colored green; in units of $\tau _{w}^{-1}$). The change in response to pattern 1 (2) is indicated by a solid (dashed) curve (as in panel a.i). At lower inhibition synaptic potentiation caused by one pattern cancels against synaptic depression caused by the other; at higher inhibition levels both are zero. **b**. Stable solution of standard BCM and weight dependent BCM as a function of stimulus parameter $\phi$ (post synaptic activities in top panel; synaptic weights in bottom panel). As $\phi$ increases, the angle between stimulus vectors decreases (become more parallel). For standard BCM the weights diverge (thin red curves), while for weight dependent BCM they converge (black and green). Feedforward inhibition was fixed to $u=1$

Next we repeat the simulation with weight dependent BCM. The dynamics to reach the equilibrium are similar and the threshold oscillates a bit before settling down, Fig. 1d (right). However, the stable fixed points are different. The plasticity is still competitive as one stimulus is randomly preferred above the equivalent other stimulus. However, the post-synaptic response to the losing stimulus remains above zero, therefore competition in weight dependent BCM model is weaker than in standard BCM. In parallel, the synaptic weights are less extreme, Fig. 1d (right, bottom). This raises the question if competition is always less in the weight dependent BCM model. We systematically varied the inhibition and examine the weights in steady state, that is, at the end of a long simulation, Fig. 2a. One can distinguish two regimes.

For strong inhibition (right region, above $u\gtrsim 2$), we retrieve standard BCM behavior. The neuron shows winner-take-all selectivity and weights are as expected from standard BCM. There is no plasticity for either stimulus at equilibrium, $\Delta \varvec{w}^{(1)}=\Delta \varvec{w}^{(2)}=\varvec{0}$.

However, for weak inhibition (left region in Fig. 2a) the neuron is always activated by both patterns, but not to the same extent. The selectivity depends on the amount of inhibition. The stronger the inhibition, the stronger the selectivity. Note however that competition is even present at zero inhibition, which is unlike competition resulting from lateral inhibition commonly used in unsupervised plasticity models. Below we define selectivity mathematically (Eq. (11)).

The nature of the stable fixed points in this regime is different from standard BCM. The bottom panel in Fig. 2a shows the weight update per stimulus and reveals that in weight dependent BCM the synaptic change in response to one pattern is canceled by that of the other pattern, $\Delta \varvec{w}^{(2)}=-\Delta \varvec{w}^{(1)}$. The weights keep changing and only the net change is zero, $\langle \Delta \varvec{w}\rangle =\Delta \varvec{w}^{(2)}+\Delta \varvec{w}^{(1)}=\varvec{0}$.

One can wonder if the weak competition is due to the constraint on the non-negativity of the excitatory synapses. However, while one excitatory weight can come close to zero, it does not exactly equal zero, Fig. 2a.ii. In other words, the dynamics do not run into the $v_{i}\ge 0$ bound.

The critical level of inhibition for which weight dependent BCM solutions become identical to standard BCM depends on the stimulus. In Fig. 2b the angle between the stimuli, $\phi$, was varied, while the level of inhibition was fixed to 1. For nearly orthogonal stimuli ($\phi \approx 0$) the solutions of standard and weight dependent BCM were identical. However, for nearly parallel stimuli ($\phi \rightarrow \pi /2$) the weights in standard BCM diverge to ensure that the activity remains zero for one stimulus and nonzero for the other, while in weight dependent BCM the weakly competitive solution is stable.

#### Effect of neural noise

We first examined whether the above results are robust to noise. We denote the noisy version of the post-synaptic activity as $\tilde{y}=y+\nu _{y}$ where $\nu _{y}$ is zero-mean Gaussian noise added to the output of the neuron with variance $\sigma _{y}^{2}$ (noise added to the input had similar effects). In order to not change the mean activity and concentrate solely on the effect of noise, we assume a linear input-output relation $y=\varvec{w.x}$. With added noise the averaged modification function in standard BCM becomes

**Fig. 3:**
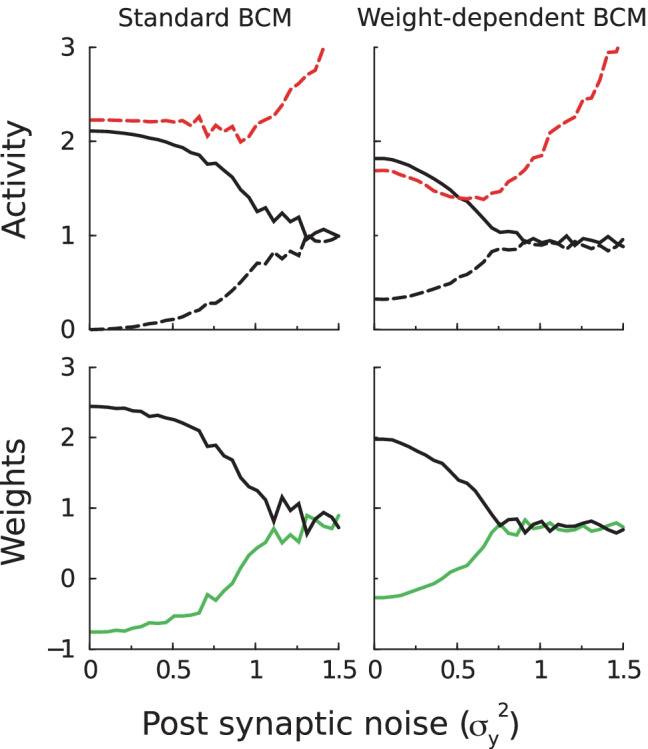
In both standard BCM and weight dependent BCM post-synaptic noise weakens competition. Top panel: the activities in response to either stimulus and the threshold (red dashed) after the plasticity has converged. Bottom: panel: the (effective) synaptic weights. Stimulus as in Fig. 1

5 $$\begin{aligned} \langle \Delta w_{i}\rangle&\propto \int x_{i}F(y)\mathcal{\mathcal{N}}(0,\sigma _{y}^{2})d\nu _{y}\\&=x_{i}\langle \tilde{y}(\tilde{y}-\theta )\rangle \\&=x_{i}[y\left( y-\theta \right) +\sigma _{y}^{2}] \end{aligned}$$

Similarly the threshold becomes $\theta =\frac{1}{K}\sum _{k}(\widetilde{y}^{k})^{2}=\frac{1}{K}\sum _{k}(y^{k})^{2}+\sigma _{y}^{2}$, where we used that for large enough $\tau _{\theta }$, threshold $\theta$ and activity $\tilde{y}$ are uncorrelated. Solving for $\langle \Delta \varvec{w}\rangle =\varvec{0}$ as above yields the equilibria. For small noise one has $y^{k}(y^{k}-\theta )+\sigma _{y}^{2}=0$ for both *k*, which combined together with the threshold equation yields $y^{1}+y^{2}=2$. This then gives $(y^{1}-1)[(y^{1})^{2}-2y^{1}+\sigma _{y}^{2}]=0.$ As the $y^{1}=y^{2}=1$ solution is unstable, one has $(y^{1})^{2}-2y^{1}+\sigma _{y}^{2}=0$, or

$$\begin{aligned} y^{1,2}&=1\pm \sqrt{1-\sigma _{y}^{2}}\\ \theta&=2 \end{aligned}$$

For large noise ($\sigma _{y}\ge 1$), one has $y^{1}=y^{2}=1$ and $\theta =1+\sigma _{y}^{2}$. The noise thus reduces the competition between the stimuli, and at high noise levels the fixed points collapse into one single, symmetric fixed point ($\varvec{w}\propto \varvec{1}$ for the stimulus used). Indeed this is what we find in simulation, Fig. 3b(left). In the simulation of weight dependent BCM, we see a similar effect of the noise, Fig. 3b(right). In summary, in both standard BCM and weight dependent BCM noise reduces the competition.

### Phase-plane analysis

**Fig. 4:**
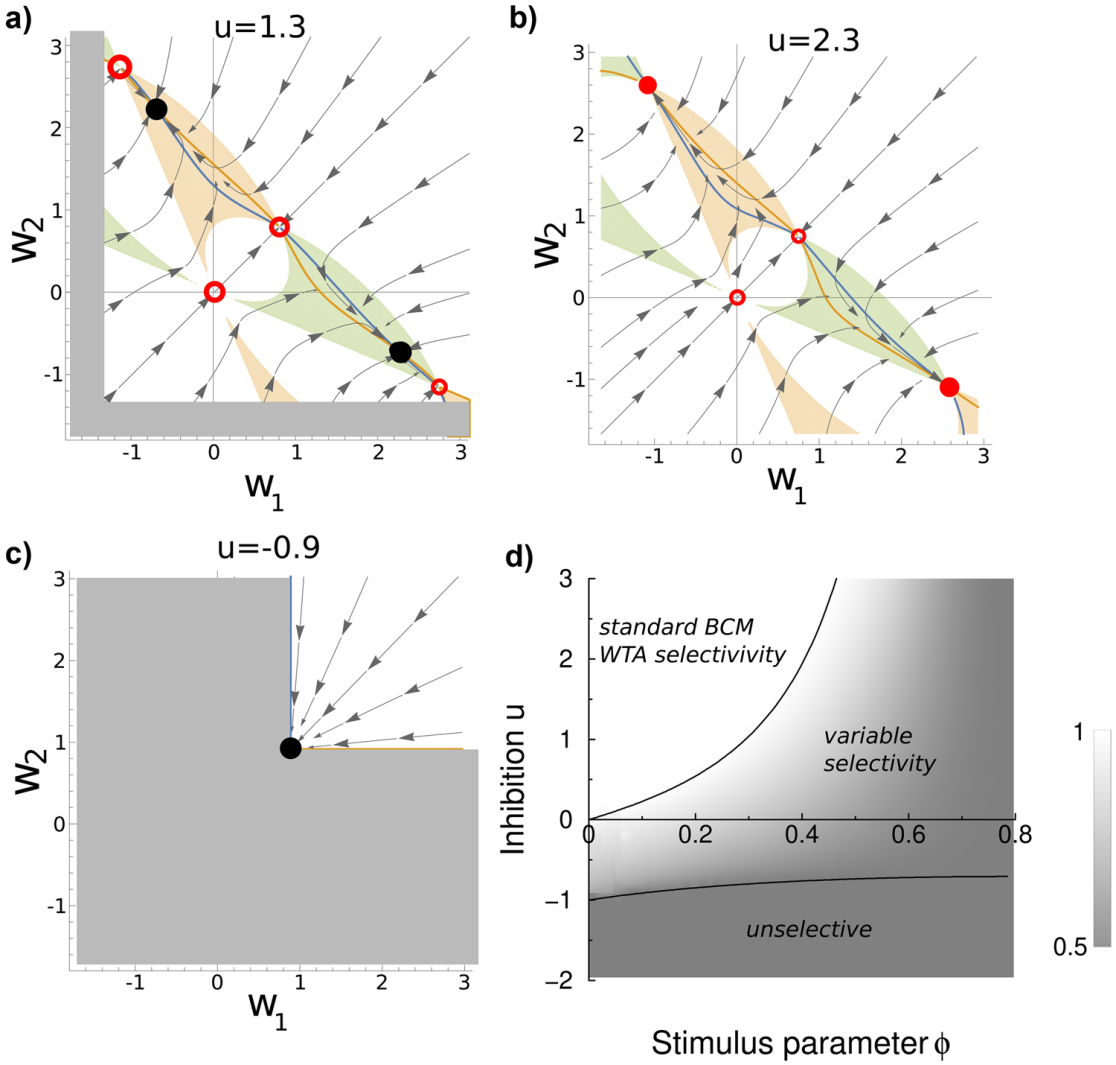
Dependence of stable fixed points of weight dependent BCM in two dimensions on inhibition. a) Phase portraits of the weights at weak ($u=1.3$) and b) strong inhibition ($u=2.3$). The null-clines ($dw_{1}/dt=0$ in blue, and $dw_{2}/dt=0$ in orange) intersect five times. The stable (unstable) fixed points are indicated with filled (open) circles. For weak inhibition, the fixed points of standard BCM (the top-left and bottom right red circles) are unstable, and instead new stable fixed points arise (black circles). The tan colored regions indicate where the first input pattern leads to depression and the second stimulus leads to potentiation (and the reverse for the green region). The new fixed points are always in these regions. In the grey region the excitatory weight would need to become negative, breaking our assumptions. b) For strong inhibition, there are only four FPs. The top-left and bottom-right fixed points from standard BCM are now stable. The symmetric fixed points, around $\varvec{w}=(0,0)$ and $\varvec{w}\approx (1,1)$ are always unstable. The grey restricted region ($w_{1,2}<-2.3$) falls out of view. c) When the neuron receives fixed feed-forward excitation instead of feed-forward inhibition (negative *u*), only a symmetric fixed point remains. d) Phase diagram of the stable fixed points of weight dependent BCM as a function the inhibition strength *u* and the stimulus parameter $\phi$. The grey-level indicates the selectivity *s*, Eq. (11). Allowing for feedforward excitation, there are three types of fixed points: classical BCM with winner-take-all selectivity ($s=1$), fixed points unique to weight dependent BCM with selectivity set by the level of inhibition($0<s<1$), and unselective fixed points ($s=0$)

To better understand the behaviour of weight dependent BCM in the regimes of both weak and strong inhibition we study the $(w_{1},w_{2})$-plane, Fig. 4a+b. We are mainly interested in the fixed points, which are given by

6 $$\begin{aligned} \begin{aligned} \Delta w_{1}^{(1)}(w_{1,}w_{2})+\Delta w_{1}^{(2)}(w_{1,}w_{2})&=0\\ \Delta w_{2}^{(1)}(w_{1,}w_{2})+\Delta w_{2}^{(2)}(w_{1,}w_{2})&=0 \end{aligned} \end{aligned}$$

The fixed points can be found from the null-clines. The null-clines are the curves at which the net change of either weight is zero (blue and orange curves). The fixed points (FPs) are located where the null-clines intersect. For weak inhibition ($u=1.3$) there are five such intersections, Fig. 4a, circles. In addition, there is an unstable FP for $w_{1}=w_{2}=0$. The fixed points present in standard BCM remain present in weight dependent BCM (indicated in red). This is easy to see, because if $y=0$ or $y=\theta$ in Eq. (3), then $\Delta w=0$. However, in addition two new FPs arise, unique to weight dependent BCM.

We numerically determined the stability from the Jacobian using standard linear stability analysis. At the standard BCM FPs, however, the update function is not differentiable (see Fig. 1b), and the Jacobian is not well-defined. However, we can use the stronger requirement that the FP is stable, if it is stable for both for depression and for potentiation cases. The eigenvalues of the Jacobian need to be negative for both piecewise continuous functions on either side of the fixed point. The resulting stable FPs are indicated by solid circles, the unstable ones by open circles.

Note that when inhibition is very small, the synaptic weights associated to the standard BCM fixed points could become inaccessible, because they would require a negative excitatory weight ($w_{i}<-u$). This restricted region is indicated in grey. However, from about $u\gtrsim 0.5$ (for these stimuli), the fixed points are accessible but unstable in the weight dependent model.

For strong inhibition ($u=2.3$) a qualitatively different situation occurs. The new FPs and those from standard BCM merge; the null-clines intersect now only three times, compare Fig. 4b to Fig. 4a. The stable fixed points of weight dependent BCM are identical to the standard BCM fixed points in the strong inhibition regime, as in our simulations.

#### Critical amount of inhibition

We have seen that above a certain level of inhibition the weight dependent model behaves identically to standard BCM. Here we calculate the amount of inhibition at which this transition occurs.

First, we determine where in the $w-$plane fixed points could occur. The new fixed points require that one stimulus leads to potentiation and the other to depression. In order words, $y^{(1)}>\theta$ and $y^{(2)}<\theta$, or vice versa. In *y*-space the region where $y^{(1)}>\theta$, where $\theta =\frac{1}{2}\sum _{k=1,2}\left[ y^{(k)}\right] ^{2}$, is given by a disc of unit radius, centered at $y^{(1)}=1$, $y^{(2)}=0$, and similar for the alternative case. Because the post-synaptic activation is given by $y^{(k)}=(X\varvec{w})_{k}$, the corresponding regions in *w*-space are found by the linear transform $$\varvec{w}=X^{-1}\left( \begin{array}{c} y^{(1)}\\ y^{(2)} \end{array}\right)$$. These regions are indicated by the light green and pink shading in Fig. 4a+b The new fixed points must lie inside them. Meanwhile the standard BCM fixed points must lie on the edge of these regions as for standard BCM $y^{(k)}=\theta$. Secondary regions emerge corresponding to the case where the post-synaptic activity becomes negative for one stimulus (bottom-left light green and pink regions). However, there are no null-clines in these regions and hence there are no FPs.

Because the stimulus components are assumed positive ($x_{i}^{(k)}\ge 0$), the sign of the plasticity is determined only by the post-synaptic activity. For a given stimulus the synapses either all undergo potentiation or all undergo depression. Mix cases do not occur, simplifying the analysis. Assume for now that the first stimulus leads to depression of all synapses, and the second stimulus leads to potentiation. From Eq. (3) the FPs can be written as a matrix equation

7 $$\begin{aligned} \left( \begin{array}{cc} x_{1}^{(1)}(w_{1}+u) &{} x_{1}^{(2)}\\ x_{2}^{(1)}(w_{2}+u) &{} x_{2}^{(2)} \end{array}\right) \left( \begin{array}{c} F(y^{1})\\ F(y^{2}) \end{array}\right) =\varvec{0} \end{aligned}$$

The standard BCM FPs correspond to $F(y^{1})=F(y^{2})=0.$ The new solution(s) for which $F(y^{k})\ne 0$ requires that the determinant of the matrix is zero, i.e.

8 $$\begin{aligned} x_{1}^{(1)}x_{2}^{(2)}(w_{1}+u)=x_{1}^{(2)}x_{2}^{(1)}(w_{2}+u) \end{aligned}$$

In other words, the possible fixed points must lie on a line in the $(w_{1},w_{2})$ plane. This corresponds to the top-left fixed point (black circle).

Another, mirrored solution occurs when instead the first stimulus leads to potentiation and the second stimulus leads to depression (the lower right fixed point). In that case $x_{2}^{(1)}x_{1}^{(2)}(w_{1}+u)=x_{1}^{(1)}x_{2}^{(2)}(w_{2}+u)$. Both lines go through the point $(w_{1},w_{2})=(-u,-u)$.

This reduction has two applications. First, one can now eliminate $w_{2}$ and express $\Delta w_{1}(w_{1},w_{2})$ as a quartic polynomial in $w_{1}$, with *u* and *x* as a parameters. The polynomial has highly complicated coefficients, caused by the dependence of $\theta$ on $\varvec{w}$. Nevertheless, this reduction simplifies numerical solution of the fixed points to a one dimensional equation. Together with the condition that the first stimulus indeed leads to depression, numerical solution of this polynomial confirmed our simulation results: There is at most one solution, and, when inhibition is strong the standard BCM fixed points are stable and there are no additional fixed points.

Second, the above analysis also yields the critical amount of inhibition above which the FPs merge and the standard BCM solutions become stable. As seen from Fig. 2a at the transition point $\Delta w^{(1)}=\Delta w^{(2)}=0$. At this point the weights are both standard BCM fixed points (Eq. (4)) but also must fall on the line given by Eq. (8).

Eq. (8) can be re-written as $u=(-x_{1}^{(1)}x_{2}^{(2)}w_{1}+x_{1}^{(2)}x_{2}^{(1)}w_{2})/\det X$. Using that the top-left standard BCM fixed point is given by $\varvec{w}=(-2x_{2}^{(1)},2x_{1}^{(1)})/\det X$, this yields the critical level of inhibition

9 $$\begin{aligned} u^{*}=2\frac{x_{1}^{(1)}x_{2}^{(1)}\left[ x_{1}^{(2)}+x_{2}^{(2)}\right] }{\left[ x_{1}^{(1)}x_{2}^{(2)}-x_{1}^{(2)}x_{2}^{(1)}\right] ^{2}} \end{aligned}$$

(and similar equation with superscript (1) and (2) swapped for the case that the first stimulus leads to potentiation). When the feed-forward inhibition exceeds this level ($u>u^{*}$), the standard BCM FPs are stable. For non-symmetric stimuli the levels of critical inhibition will be different for each fixed point.

For completeness we can extend this analysis to negative *u*. In that case the neuron receives static feed-forward *excitation*. In order to obtain low enough activity the weights now reach the lower bound $w_{i}=-u$, Fig. 4c. The critical value of inhibition for this to happen can be found by substitution of $\varvec{w}=(-u,-u)$ in $y=\theta$, yielding

10 $$\begin{aligned} u^{**}=-2\frac{x_{1}^{(2)}+x_{2}^{(2)}}{\left[ x_{1}^{(1)}+x_{2}^{(1)}\right] ^{2}+\left[ x_{1}^{(2)}+x_{2}^{(2)}\right] ^{2}} \end{aligned}$$

At and below this value of inhibition the weights are $\varvec{w}=(-u,-u)$ and the neuron is completely non-selective.

The regimes are summarized in Fig. 4d for our specific stimulus parametrization where $$u^{*}=2\sin 2\phi /[\cos \phi +\cos 3\phi +\sin \phi -\sin 3\phi ]$$, and $$u^{**}=-[\sqrt{2}\sin (\phi +\pi /4)]^{-1}$$. The grey-level indicates the selectivity which is defined as

11 $$\begin{aligned} s\equiv \frac{\max _{k}y^{k}}{\sum _{l}y^{l}} \end{aligned}$$

It ranges from 1/2 for the unselective case (dark grey) to 1 for standard BCM (white).

## Receptive field development with many inputs

Next, we analyzed how the above observations carry over to higher dimensional situations. We used a neuron with $N=20$ inputs receiving $K=20$ stimuli which had all the same spatial profile but had different centers. We had noted earlier that for stimuli that are smooth, such as von Mises shapes $x_{i}^{(k)}\sim \exp [-(1+\cos 2\pi \frac{i-k}{N})]$, the convergence of standard BCM becomes exponentially slow as *N* increases (Froc & van Rossum, 2019). Hence we used triangular shaped stimuli, $x_{i}^{(k)}=\left[ 1-\frac{2}{N\omega }|i-k|\right] _{+}$, where $|i-k|$ is taking periodic boundaries into account, and $\omega$ is a parameter setting the width of the profile (set to 0.5). Stimuli were presented in a randomly permuted, fixed sequence.

**Fig. 5:**
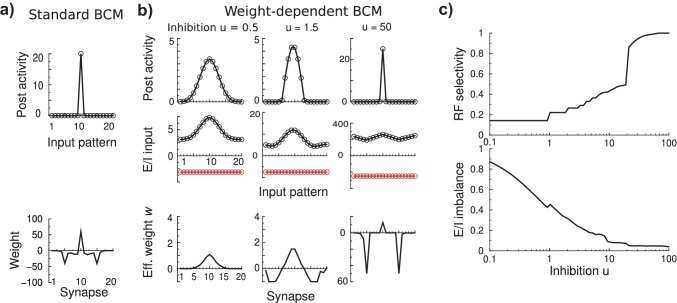
BCM in higher dimensions behaves similarly to two dimensions. Simulation of a single neuron with $N=20$ inputs, trained on $K=20$ triangular stimuli, width $\omega =0.5$. **a**) Top panel: The activity for a given input pattern, that is the convolution of the input patterns with the weights, showing that it is active only in response to one of the input patterns. Plots were re-centered such that the central synapse was the strongest. Bottom panel: Standard BCM yields an oscillating weight profile. **b**) In weight dependent BCM the receptive fields and the shape of the weight profile depend on the strength of feed-forward inhibition. The stronger the inhibition, the more selective. At very strong inhibition one retrieves a solution similar to standard BCM. The middle panels show the total excitatory (black) and inhibitory (red) input for each stimulus; the bottom panel show the weight profile. Note the changes in y-axis scale across panels. **c**) Top: The selectivity of the neuron trained with weight dependent BCM. When equal to 1, only one stimulus activates the neuron. Bottom: The excitation/inhibition imbalance expressed the fraction of excess excitation for the preferred stimulus that is not canceled against inhibition and drives the neuron. For strong inhibition, most excitation is canceled by the inhibitory drive

In the case of standard BCM, when there are $K=N$ stimulus patterns that span the *N*-dimensional space (i.e. the stimulus vectors are linearly independent), the winner-take-all solution carries over from the 2D case. In the steady state the neuron becomes selective to one stimulus only, and remains silent in response to all other stimuli (Castellani et al., 1999). The resulting weights have an oscillatory character, Fig. 5a. This is a direct consequence of the strong selectivity: if the neuron is only active in response to one stimulus, the synaptic weights need to be arranged such that for all other stimuli the inputs exactly cancel each other. The fixed points generalize from the 2D case.

12 $$\begin{aligned} w_{i}=K(X^{-1})_{i,m} \end{aligned}$$

For weight dependent BCM the above approach eliminating one of the weights is in principle extendable to higher dimensions. However, as soon as more than one stimulus leads to depression, the condition on the determinant in Eq. (7) includes products of elements of $\varvec{w}$ and the reduction is no longer linear. Furthermore, the enumeration of all possible cases becomes cumbersome. Hence, we rely on simulations only.

We find that for weak feedforward inhibition all effective weights are positive and the neuron responds to all stimuli, albeit at different rates. It is less selective, Fig. 5b(left). As inhibition is increased, negative effective weights arise and the neuron responds only to some stimuli. As an aside, with our over-simplified threshold-linear neuron we retrieve contrast-invariant tuning curves well-known from V1 physiology. Finally for strong inhibition, the neuron becomes selective to a single stimulus, similar to the case in standard BCM.

To further characterize these regimes, we plot the selectivity Eq. (11) which now ranges from 1/*K* for non-selective neurons, to 1 for winner-take-all competition. This selectivity increases with increasing inhibition, Fig. 5c, top. A step-like pattern can be seen as less and less stimuli yield a response as inhibition is increased.

As the inhibition increases, the neuron receives more excitation but almost all of it is canceled by inhibition. To quantify this we calculate the imbalance as the amount of excess excitation at the peak response. We define it as $m\equiv [\max _{k}E_{k}-I]/\max E_{k}$, where $E_{k}$ is the excitatory current for stimulus *k*, $E_{k}=\sum _{i}w_{i}x_{i}^{(k)}$, and $I=u\sum _{i}x_{i}^{(k)}$ the inhibitory current which is independent of stimulus *k* for this stimulus ensemble. Note that unlike other classic balanced models, the neuron is still mean-driven and not noise-driven. The imbalance ranges from 1 when the neuron is exclusively driven by excitation, and decreases when the inhibition cancels the excitation. In the highly selective regime the excitation and inhibition largely cancel against each other. In summary, also when considering neurons with multiple inputs, weight dependent BCM develops receptive fields where the feedforward inhibition determines the selectivity.

### V1 receptive field development

To examine whether the weight dependent BCM model would be appropriate as a model for sensory cortex development, we examined a neuron trained with natural image patches. We trained the neuron with 40000 randomly selected circular shape patches of 400 pixels of natural images taken from Hyvärinen et al. (2009). Retinal pre-processing was modeled as a balanced Difference-of-Gaussians filter with a center width of 1 pixel and a surround of 3 pixels (Law & Cooper, 1994). To prevent negative *x*, the pixel intensities $x_{i}$ were scaled and offset to range from 0 to 1. Not only are the input patterns now much less structured than above, the number of input patterns is much larger than the number of inputs ($K\gg N$). We cycled through the patches until equilibrium was reached.

**Fig. 6:**
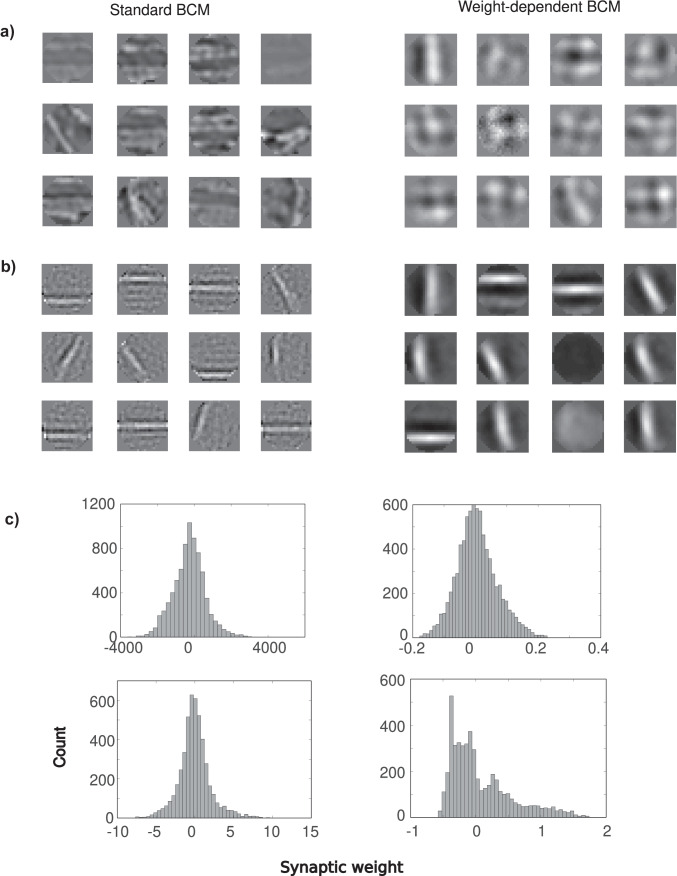
Receptive field development. **a**) Receptive field development under standard BCM (left) and weight dependent BCM (right, $u=1$). The neuron is trained with 40000 random circular natural image patches and had a linear rectifying non-linearity. Inputs ranged between 0 and 1. Weight vectors are shown. **b**) As in a) but the input patterns were made zero mean and the neuron had a sigmoid non-linearity. This results in localized weight profiles, resembling the sparse Gabor-like receptive fields found in primary visual cortex. **c**) Corresponding synaptic weight histograms of panels a) and b) pooled over all samples. Standard BCM tends to lead to synaptic weights that are much larger in magnitude. For zero mean inputs (bottom panels) the spread in the distributions is comparable, but weight dependent BCM has a positively skewed distribution

Without further modification however, standard BCM leads again to receptive fields that are highly selective, with typically only a few stimuli giving a strong response. As a result the weights have a lot of fine structure, Fig. 6a left. The synaptic weights are again large in magnitude, Fig. 6c, cf. Fig. 5. For weight dependent BCM, the receptive fields are again much less selective at this intermediate level of inhibition and the weight profiles are smooth, Fig. 6a right. The corresponding synaptic weights, Fig. 6c, follow again a smooth distribution, centered around zero, but with a much smaller variance.

Neither variant of BCM leads to localized Gabor-type receptive fields such as one sees in primary visual cortex. This is not surprising. It is known that in order for standard BCM plasticity to yield such receptive fields, it needs to be modified. First, the input patterns need to be made zero-mean (Blais et al., 1998). Second, the post-synaptic activity needs to be a non-linear function of the input. As in (Blais et al., 1998) we used a saturating sigmoid $y(h)=\sigma _{-}\tanh (h/\sigma _{-})$ when the net input $h=\varvec{w} \cdot \varvec{x}$ was negative and $y(h)=\sigma _{+}\tanh (h/\sigma _{+})$ when *h* was positive. As a result the neuron is linear ($y\approx h$) for small inputs, but saturates so that $-\sigma _{-}<y<\sigma _{+}$, where $\sigma _{-}=0.01$ and $\sigma _{+}=50$.

Together, these modifications ensure that the plasticity becomes sensitive to higher order moments in the input data, which are crucial in developing localized receptive fields, but also generally sufficient. Many models with Hebbian learning and an output non-linearity yield localized receptive fields, provided the input is whitened (Brito & Gerstner, 2016). When we repeat the simulations with these modifications, both standard and weight dependent BCM yield Gabor-like weight profiles, Fig. 6b. Thus while both BCM variants can yield localized receptive fields, they only do so under specific conditions.

While the distribution of synaptic weights now has a comparable spread and roughly similar shape, it becomes positively skewed in the case of weight dependent BCM, resembling observed weight distribution, Fig. 6c.

## Discussion

In summary, we have introduced a variant of BCM which in two aspects differs from standard BCM. First, the weights coming into a neuron are split into in inhibitory and excitatory ones. This is a well-known construction in computational neuroscience which allows us to incorporate the second aspect, namely weight dependence of excitatory plasticity. Experimentally, weight dependence of plasticity is reasonably well established, in particular for the potentiation branch. Far less papers studied weight dependence in synaptic depression, yet we are not aware of any study disputing the findings on which our model is based. Indeed, it is difficult to imagine how despite numerous non-linearities and saturating processes such as receptor insertion, plasticity could be independent of the current synaptic weight.

The inclusion of weight dependence in unsupervised plasticity has been used to abolish the need for hard bounds on synaptic weights, and stabilize learning (van Rossum et al., 2000; Rubin et al., 2001; Gütig et al., 2003; Morrison et al., 2007; Humble et al., 2019; van Rossum et al., 2012). In STDP models, as weights grow as a result of potentiation inducing pre/post spike time pairs, they are eventually knocked down again by strong depression inducing spike-pairs. The weight dependent BCM model prevents synaptic weights from running away to negative values, because depression becomes weaker for small weights ($\Delta v_{i}\propto v_{i}$) (also see text below Eq. (3)). However, because the current model is not stochastic, a large weight might never experience depression. As a result, weight dependence, which yields extra stability in STDP, here does not obviate the adaptation of the BCM threshold.

While weight dependent BCM does not appear to change the number of stable fixed points or the overall learning dynamics, it introduces an additional parameter. This parameter - the strength of feed-forward inhibition - sets the amount of competition. Competition is less selective in the weight dependent BCM model when the inhibition is weak; when inhibition is stronger than a certain threshold value, we retrieve standard BCM-like winner-take-all competition. It would interesting to measure the competition experimentally, ideally using an isolated neuron. We speculate that biology might have exploited this to develop receptive fields with different amounts of selectivity, e.g. create visual receptive fields with different tuning widths (Fig. 5).

In regards to the formation of sparse receptive fields, the weight dependent model seems to have little benefit over standard BCM. Both require zero-mean input. While such a pre-processing requirement is common in these type of models, the biology of it is unclear. From our results it seems that fixed feed-forward inhibition does not suffice for this purpose (Fig. 6a).

Competition and selectivity among inputs are generally seen as essential ingredients in networks that perform sensory encoding. So one could wonder if strong competition is preferable from a functional point of view. For individual, isolated neurons there is no need to strongly select specific input patterns at the expense of others inputs as occurs in standard BCM. Competition could arise on the network level only, for instance through* lateral *inhibition, while single neurons do not need to be strongly selective (Hertz et al., 1991; Billings & van Rossum, 2009).

We assumed that the plasticity occurs exclusively in the excitatory connections and that the feed-forward inhibition is fixed. Knowledge of the inhibitory plasticity and its weight dependence is still scarce, but as experiments progress inhibitory plasticity can be included in the model. Another extension is to consider this modified plasticity rule in recurrent networks.

## APPENDIX: Relation to STDP plasticity

We examine two possible ways to derive weight dependent BCM from weight dependent spike timing dependent plasticity (STDP), and how they lead to different weight dependence.

Based on Shouval et al. (2002a), Graupner and Brunel (2012) introduced a STPD model in which, whenever a hypothesized post-synaptic calcium concentration exceeds a high threshold, potentiation occurs, but when it only reaches a certain lower threshold, depression results. Sub-cellular cascades could easily implement such behavior. This model can fit a remarkably large number of STDP data sets. Weight-dependence is easily included in this model: calcium determines whether the synapse is potentiated or depressed, and when it is depressed it should do so proportionally to the weight. When this STDP rule is tuned to give BCM-like plasticity (see Graupner & Brunel, 2012), inclusion of weight dependence in STDP directly leads to the weight dependent BCM model used here.

An alternative way to derive a BCM-like modification curve from STDP assumes that the net modification of the synapse is the sum of both the potentiation and the depression term. Izhikevich and Desai (2003) restricted the plasticity to nearest neighbor pre/post spike pairs only and exploited the fact that experimentally the STDP potentiation time-window tends to be narrower more pronounced than the depression window. Because at high frequencies short intervals between pre- and post-synaptic spike are more common potentiation dominates at high pairing frequencies, while at low frequencies synaptic depression dominates. The sum of potentiation and depression mimicks the BCM modification curve.

Including weight dependence of the depression component yields for the modification per pre-synaptic input spike

13 $$\begin{aligned} \Delta w=f\left( \frac{A_{+}}{1/\tau _{+}+f}+\frac{(w+u)A_{-}}{1/\tau _{-}+f}\right) \end{aligned}$$

where *f* is the post-synaptic Poisson rate. The first right hand side term is the potentiation part, the second the depression. To obtain a BCM-like modification curve, the LTD and LTP amplitudes $A_{-}$ and $A_{+}$, need to satisfy $A_{-}<0$, $A_{+}>|A_{-}|$ and $A_{+}\tau _{-}<|A_{-}|\tau _{-}$.

It can been seen from Eq. (13) that a larger weight experiences stronger depression, but that it also has a higher potentiation threshold. Thus in contrast to Fig. 1b, the zero-crossing of the modification curve shifts with the weight. Because, in contrast to the Graupner and Brunel model, in this model both LTD and LTP need to be biophysically expressed for every spike pair to obtain the BCM curve, we think that this is biophysically less likely, for instance due to the high metabolic costs of plasticity (Li & Van Rossum, 2020). It would be an interesting electrophysiological experiment to decide whether the BCM threshold indeed depends on individual synaptic weight; we have not examined this model variant in detail.
